# Online Patient Recruitment in Clinical Trials: Systematic Review and Meta-Analysis

**DOI:** 10.2196/22179

**Published:** 2020-11-04

**Authors:** Mette Brøgger-Mikkelsen, Zarqa Ali, John R Zibert, Anders Daniel Andersen, Simon Francis Thomsen

**Affiliations:** 1 Department of Dermatology Bispebjerg Hospital Copenhagen Denmark; 2 Department of Biomedical Sciences University of Copenhagen Copenhagen Denmark; 3 Studies&Me A/S LEO Innovation Lab Copenhagen Denmark

**Keywords:** online clinical trial, web-based clinical trial, hybrid clinical trial, online recruitment, remote recruitment, recruitment, clinical trial, conversion rate

## Abstract

**Background:**

Recruitment for clinical trials continues to be a challenge, as patient recruitment is the single biggest cause of trial delays. Around 80% of trials fail to meet the initial enrollment target and timeline, and these delays can result in lost revenue of as much as US $8 million per day for drug developing companies.

**Objective:**

This study aimed to conduct a systematic review and meta-analysis examining the effectiveness of online recruitment of participants for clinical trials compared with traditional in-clinic/offline recruitment methods.

**Methods:**

Data on recruitment rates (the average number of patients enrolled in the study per month and per day of active recruitment) and conversion rates (the percentage of participants screened who proceed to enroll into the clinical trial), as well as study characteristics and patient demographics were collected from the included studies. Differences in online and offline recruitment rates and conversion rates were examined using random effects models. Further, a nonparametric paired Wilcoxon test was used for additional analysis on the cost-effectiveness of online patient recruitment. All data analyses were conducted in R language, and *P*<.05 was considered significant.

**Results:**

In total, 3861 articles were screened for inclusion. Of these, 61 studies were included in the review, and 23 of these were further included in the meta-analysis. We found online recruitment to be significantly more effective with respect to the recruitment rate for active days of recruitment, where 100% (7/7) of the studies included had a better online recruitment rate compared with offline recruitment (incidence rate ratio [IRR] 4.17, *P*=.04). When examining the entire recruitment period in months we found that 52% (12/23) of the studies had a better online recruitment rate compared with the offline recruitment rate (IRR 1.11, *P*=.71). For cost-effectiveness, we found that online recruitment had a significantly lower cost per enrollee compared with offline recruitment (US $72 vs US $199, *P*=.04). Finally, we found that 69% (9/13) of studies had significantly better offline conversion rates compared with online conversion rates (risk ratio 0.8, *P=*.02).

**Conclusions:**

Targeting potential participants using online remedies is an effective approach for patient recruitment for clinical research. Online recruitment was both superior in regard to time efficiency and cost-effectiveness compared with offline recruitment. In contrast, offline recruitment outperformed online recruitment with respect to conversion rate.

## Introduction

Historically, recruitment of participants for clinical trials has been critically dependent upon physician referrals and overall site performance. Increasing needs for more effective recruitment methods have led to “trial and error” models, where a number of different recruitment strategies are utilized and modified according to observed effects on recruitment [1]. Such traditional recruitment strategies include, but are not limited to, soliciting subjects through mail and telephone using health records and registers, media campaigns, newspaper advertisements, and input during radio and television talks [2].

Currently, recruitment for clinical trials continues to be a challenge, as patient recruitment is the single biggest cause of trial delays. Around 80% of trials fail to meet the initial enrollment target and timeline, and these delays can result in lost revenue of as much as US $8 million per day for drug developing companies [3]. As pointed out by Gul and Ali [4], slow and inefficient recruitment may have scientific, economic, and ethical consequences. The costs associated with recruitment are wasted, and data quality is hampered by a reduction in statistical power due to underrecruitment. Recruiting appropriate participants in a sufficient number to fulfill sample size requirements is critical for the validity of the research findings, and failure may lead to invalid or inconclusive results. Further, for traditional offline recruitment strategies, location of trial sites quickly becomes the bottleneck for participant diversity in clinical research, as sites only succeed in recruiting patients within a relatively short radius. Potentially, this results in clinical research that lacks generalizability and makes it difficult for clinical trials to be a cornerstone for providing scientific evidence on the safety and efficacy of novel pharmaceutical compounds.

Using online recruitment strategies, such as social media advertisements, Google search engine advertisements, and other website campaigns, may enable researchers to target specific study populations by demographic characteristics, location, and keywords previously used in potential participants’ user profiles. In 2018, Akers et al [5] found that Facebook advertisements gave “flexibility to monitor and modify advertisement tactics based on feedback,” and Shere et al [6] argued that social media recruitment should be redefined as an *active* recruitment tool rather than a low-budget *passive* tool, as targeting specific populations effectively yielded high recruitment rates. Similar arguments were made by Watson et al [7], Jones et al [8], and Carter-Harris et al [9] who all reported social media advertisements to be a “viable tool for more efficient and cost-effective recruitment.” The potential reach of online recruitment by far exceeds the reach that traditional recruitment methods are able to generate, but whether online recruitment strategies outperform traditional offline recruitment strategies still remains unclear in the literature. Herein, traditional in-clinic recruitment methods are referred to as offline recruitment.

The aim of this study was to evaluate the effectiveness of online patient recruitment by systematically reviewing studies that utilize online strategies for patient recruitment and by conducting meta-analyses comparing online and offline recruitment strategies on the following two recruitment metrics: recruitment rate (the number of patients enrolled in the study on average per month and per day of active recruitment) and conversion rate (the percentage of participants screened who proceed to enroll into the clinical trial). Further, this study investigated the cost-effectiveness of online recruitment compared with offline recruitment in clinical research. In the study, we investigated the following three hypotheses: (1) The recruitment rate is higher in online recruitment compared with offline recruitment; (2) The conversion rate is higher in offline recruitment compared with online recruitment; and (3) The cost per enrolled subject is lower in online recruitment compared with offline recruitment.

## Methods

### Literature Search

We conducted a systematic review and meta-analysis in accordance with the PRISMA statement [10] searching the following databases: PubMed, EMBASE, Cochrane Library, Web of Science, and Google Scholar. The search was conducted between February and May 2020, and was carried out by combining keywords within the following three topic domains: “online/remote/web-based,” “patient/participant/subject recruitment,” and “clinical trial/study.” Two reviewers (MBM and ZA) independently screened all titles and abstracts, and any discrepancies were resolved through discussion. Apart from duplicates, studies were excluded based on the content of the abstract if there was no clear indication that they investigated the feasibility of online patient recruitment.

### Screening and Study Selection

Studies were included in the systematic review provided that they fulfilled the following inclusion criteria: (1) Clinical studies using online recruitment and/or prescreening of patients for randomized controlled trials (RCTs), observational studies, and online surveys relevant to the focus of this study and (2) Clinical studies using a fully virtual approach from screening to data collection. For the meta-analysis, only studies that compared online patient recruitment with offline patient recruitment were included. Studies were excluded if they fulfilled one or more of the following exclusion criteria: (1) non-English papers; (2) systematic reviews; and (3) other (nonrelevant online programs, eg, parenting training programs).

### Data Extraction

For data collection, the first reviewer (MBM) used a structured form to extract the following qualitative and quantitative data: (1) study design and year; (2) study location by country; (3) total number of participants enrolled in the study; (4) online and offline recruitment metrics (full recruitment period in months, number of days with active recruitment, number of patients completing prescreening, number of patients enrolled by recruitment method, and costs); and (5) age and gender of the participants in the study. For studies where stated data collection was inadequate, the study authors were contacted and necessary data were obtained when possible.

### Outcomes Assessed

The primary outcome variable for analysis was an aggregate measure of recruitment effectiveness defined by the recruitment rate and conversion rate. Two analyses were carried out. First, the recruitment rate was defined as the number of patients recruited per month for the entire recruitment period, and second, the recruitment rate was defined as the number of patients recruited per day of active recruitment days. As online campaigns are mostly run in shorter periods with active online advertisements, removing the days in between advertisements was expected to provide a more realistic understanding of the recruitment. For offline recruitment, days in between nonactive recruitment were removed in the second analysis. The conversion rate was defined as the percentage of patients screened who proceeded to enroll into the clinical trial. Prescreening is either an online prescreening questionnaire, or on-ground screening or telephone call for assessing primary eligibility. If necessary, we recalculated the metrics needed for the analyses where possible.

The secondary outcome of interest was the cost-effectiveness of online recruitment compared with offline recruitment. To standardize cost data, we adjusted all costs to US$ using XE Live Exchange Rate 2020.

### Statistical Methods

We pooled effect sizes based on the Mantel-Haenszel method for both recruitment rate (incidence rate ratio [IRR]) and conversion rate (risk ratio [RR]). A random effects DerSimonian and Laird meta-analysis [11] was used to report both incidence rates and relative risks. Further, we calculated heterogeneity (DerSimonian and Laird estimator), which was examined using the *I^2^* statistics, and 95% prediction intervals were calculated. The Hartung-Knapp adjustment for a random effects model was used to calculate 95% CIs, reflecting the uncertainty in heterogeneity. All data analyses were conducted in R (R Foundation for Statistical Computing) [12] (packages included devtools, meta, dmetar, and pbkrtest), and *P*<.05 was considered significant.

### Meta-Analysis

The only criterion for carrying out the meta-analysis was the availability of sufficient outcomes. We considered that any amount of statistical heterogeneity would be acceptable, as studies included in this paper recruited for a wide range of therapeutic areas and trial interventions. Hence, we expected high heterogeneity among the included studies. However, the recruitment strategy was far more homogeneous between studies, and therefore, we considered the findings worth reporting. We performed two separate meta-analyses to determine the effectiveness of online patient recruitment.

#### Recruitment Rate

The effect size for recruitment rate was calculated as an incidence rate (IR), as this rate signifies how many events occur within a standardized timeframe. Effect sizes were pooled to generate the IRR, examining the relation between the incidence rate in the online recruitment group (IR_online_) and the one in the offline recruitment group (IR_offline_). The pooled data for recruitment rate was presented in a forest plot showcasing whether the recruitment rate was in favor of online or offline recruitment.

#### Conversion Rate

The conversion rate was calculated as an event rate with relative risk as the effect size, since event rate data deal with the number of persons experiencing an event in each group and the total sample size in each group. Effect sizes were pooled to generate RRs as the summary measure. As for recruitment rate, the pooled data were presented in a forest plot showcasing whether the conversion rate was in favor of online or offline recruitment.

### Additional Analysis

This study presents a cost-effectiveness analysis for studies included in the meta-analyses defined as cost per enrollee. As the distribution in our two paired data sets was nonnormal, cost-effectiveness was examined by a nonparametric paired sample Wilcoxon test that does not assume a specific underlying distribution of data.

### Risk of Bias

The risk of bias was assessed using the Cochrane Risk of Bias Tool for randomized studies and the ROBINS-I tool for nonrandomized studies. The studies were assessed for risk of bias in relation to our review question and not the study authors’ research question.

## Results

### Literature Search

**Figure 1:**
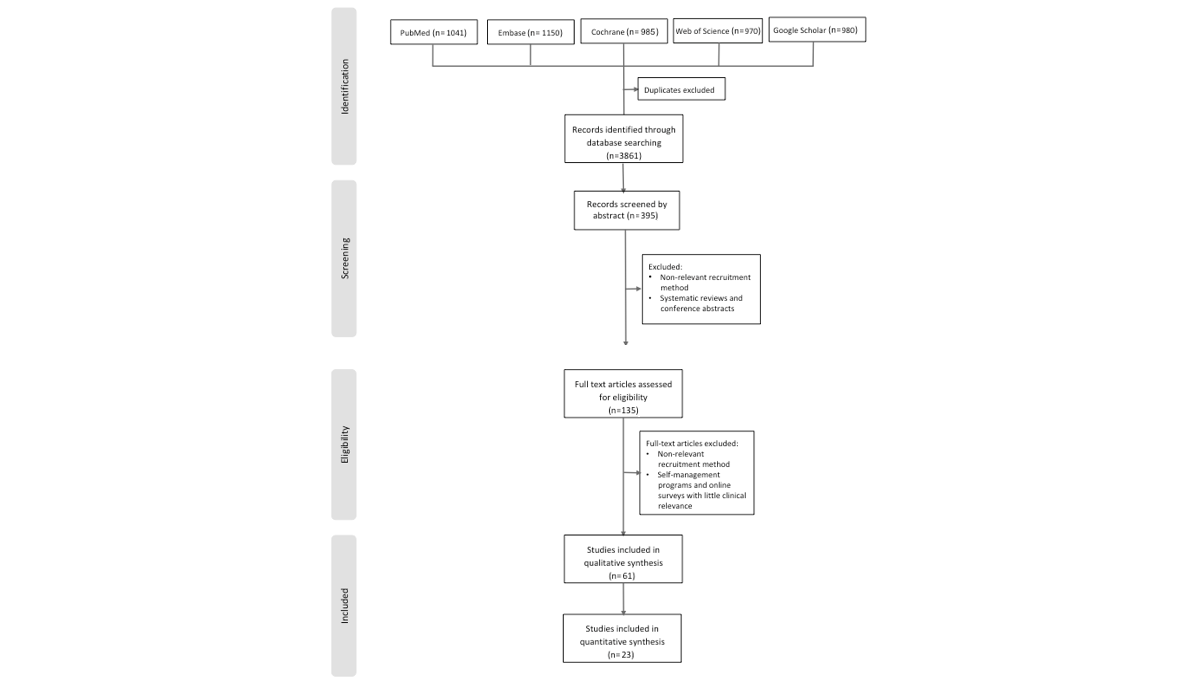
Flow diagram of the literature search.

A total of 3861 articles, including references from articles, were identified for possible inclusion based on their titles, and of these, 395 were selected for abstract screening. Of these, 135 studies were selected for full-article review, and 61 studies that reported the use of online patient recruitment without meeting any of the exclusion criteria were included in the systematic review. Of the selected studies, 23 studies investigated the feasibility of online patient recruitment compared with offline patient recruitment and were included in the meta-analysis after removing duplicates (Figure 1).

### Characteristics of All Included Studies

Multimedia Appendix 1 shows the detailed characteristics of all studies included in this review. Most were conducted in the United States or Australia, and together, the studies covered a wide range of therapeutic areas and trial interventions, with the majority of studies recruiting either adult smokers [7,9,13-24], men who have sex with men [8,25-33], or pregnant and postpartum women [6,34-39]. Of all the studies included, 15 reported a full clinical trial utilizing online recruitment itself (eg, [40-44]), while 46 of the studies described the recruitment strategy utilized in a clinical trial reported in a separate paper. In total, 39 studies covered the recruitment strategy for RCTs, 14 studies covered the recruitment strategy for observational research trials, and eight studies covered the recruitment strategy for online surveys.

#### Recruitment Strategy

Of the 61 studies included in this review, 42 studies concluded that targeting potential study participants online was an effective tool for recruitment. Only four studies reported that online recruitment was not effective [17,45-47], which might reflect the timing and time period of the online strategy in these four studies. The remaining studies did not conclude on effectiveness. A total of 55 studies used paid advertisements in their online recruitment strategy, of which 42 studies specifically used Facebook. In 2016, Adam et al [36] used Facebook advertisements to target pregnant women for an RCT and found that online strategies compared with offline strategies recruited a representative population and that recruitment rates had been “dramatically improved.” Similar findings were reported by Cowie et al [48]*,* who found that Facebook yielded highly efficient and cost-effective results when targeting people aged 60 years or older. In contrast, Rait et al [17] found that Facebook advertisements expanded reach when recruiting young adults for a smoking cessation trial; however, only a small proportion was eligible for the study, and offline methods were therefore superior to online methods both in regard to cost and time-efficiency. In total, 26 studies further used websites relevant for the specific trial, for example, popular drug control websites when recruiting for a drug abuse prevention study [34,49,50].

#### Demographics

On examining the overall patient demographics of the included studies, most papers recruited participants aged 18 years or above, and the vast majority of studies recruited both men and women. Furthermore, 46 of the studies targeted a so called *hard-to-reach* population, reaching a sample that has previously been shown to be difficult to recruit owing to either stigmatization, such as smoking cessation research (eg, [7,21]) and HIV prevention research (eg, [29,30,51-53]), or underrepresentation and low prevalence, such as research involving children with fetal alcohol spectrum disease [54] and some psychiatric conditions [55-59]. In 2014, Shere et al [6] successfully utilized social media to recruit a hard-to-reach population of women in the periconceptional period for an RCT, and among others, Morgan et al [60] effectively recruited a sample for an RCT evaluating a depression intervention through several online strategies [61].

### Characteristics of the Studies Included in the Meta-Analyses

For the meta-analyses, we included 23 studies, and all reported data on comparing the effectiveness of online and offline recruitment strategies. In total, 14 of the studies included in the meta-analyses recruited for RCTs reported in a separate paper [6-8,19-21,27,30,36,45,46,62-64]. Of the 23 studies, approximately 25% began online recruitment at a later time point than offline recruitment owing to low recruitment rates through offline recruitment approaches [6,28,36,45,62]. Data extracted from the 23 studies were used to calculate the recruitment rate, conversion rate, and cost per enrollee. Multimedia Appendix 2 shows the aggregated characteristics of the studies included in all the meta-analyses.

### Effectiveness of Online Patient Recruitment

#### Principal Findings

This study found that online recruitment strategies are superior to offline recruitment initiatives when measuring recruitment effectiveness by recruitment rate and cost-effectiveness. With online strategies, participants are recruited faster and more cost-effectively. However, this study found that offline recruitment outperforms online recruitment when converting potential participants to actual enrollees, which altogether is in line with our study hypotheses.

For recruitment rate, we found online recruitment to be significantly more effective when examining the recruitment rate for active days of recruitment, where 100% (7/7) of the studies included had a better online recruitment rate compared with the offline recruitment rate (*P*=.04). When examining the entire recruitment period in months, we found that 12 of 23 studies (52%) had a better online recruitment rate compared with the offline recruitment rate. For the conversion rate, we found that only 4 out of the 13 studies (31%) had a better online conversion rate compared with the offline conversion rate.

#### Meta-Analyses

##### Recruitment Rate

**Figure 2:**
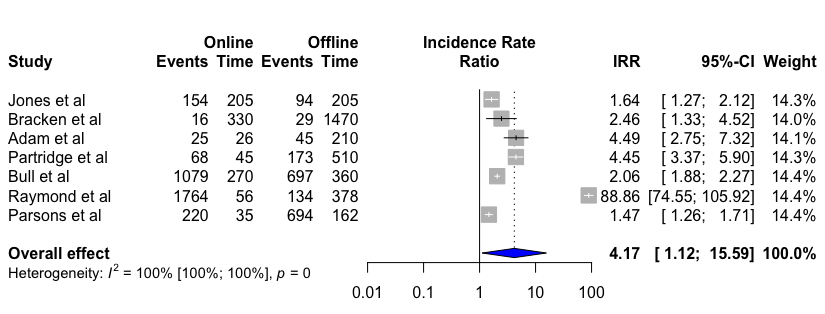
Recruitment rate for active days of enrollment for online and offline recruitment. Value <1, in favor of offline recruitment; value >1, in favor of online recruitment.

For the seven studies included in our first meta-analysis, we found that the recruitment rate for online recruitment was superior to that for traditional offline recruitment when comparing the number of active recruitment days presented as an IRR. For online recruitment, this corresponded to the number of days the advertisements were active on social media or other relevant websites. For offline recruitment, this was the number of days with active on-ground recruitment and active days of advertisements in newspapers, on busses, etc. Pooling the data from the seven studies, we found that online recruitment strategies recruited subjects significantly faster than offline recruitment strategies (IRR 4.17, 95% CI 1.12-15.59, *P*=.04). Hereby, online recruitment strategies yielded 4.17 times more participants per day of active recruitment compared with offline recruitment strategies. As expected, we found high heterogeneity between studies (*I^2^=*100%) (Figure 2).

**Figure 3:**
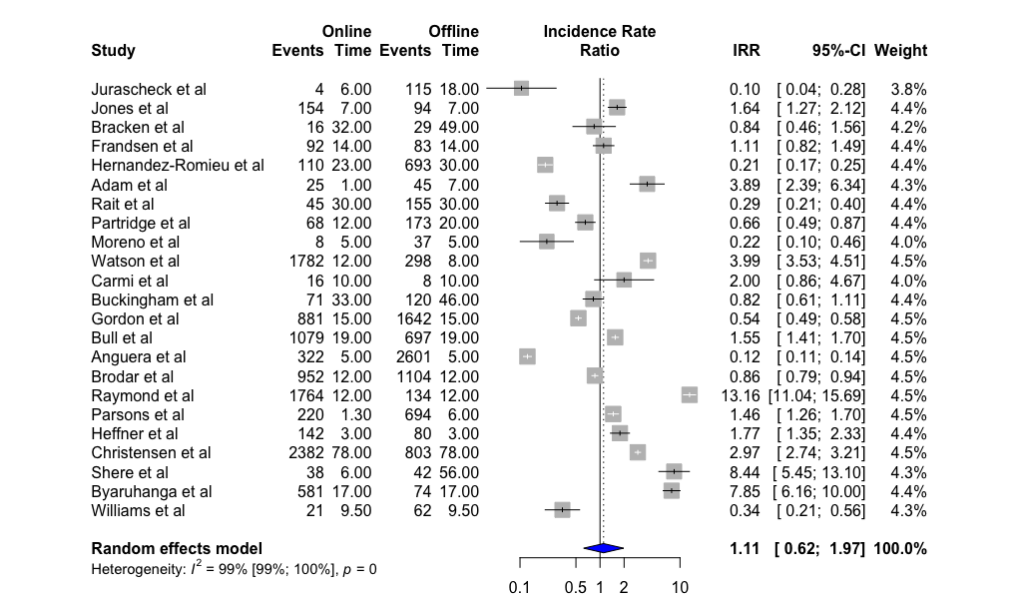
Recruitment rate for the entire period of recruitment online and offline in months. Value <1, in favor of offline recruitment; value >1, in favor of online recruitment.

For our second meta-analysis, all 23 articles were included. Here, we compared the full period of recruitment for both online and offline strategies, looking at the number of months of recruitment from the start of the first advertisement or campaign to the end of the last advertisement or campaign. Pooling the data from the 23 included studies, we found that online recruitment was similar to offline recruitment with respect to effectiveness when nonactive days within the recruitment period were also included in the analysis (IRR 1.11, 95% CI 0.62-1.97, *P*=0.7). Again, we found high heterogeneity between studies (*I^2^*=99%) (Figure 3).

##### Conversion Rate

**Figure 4:**
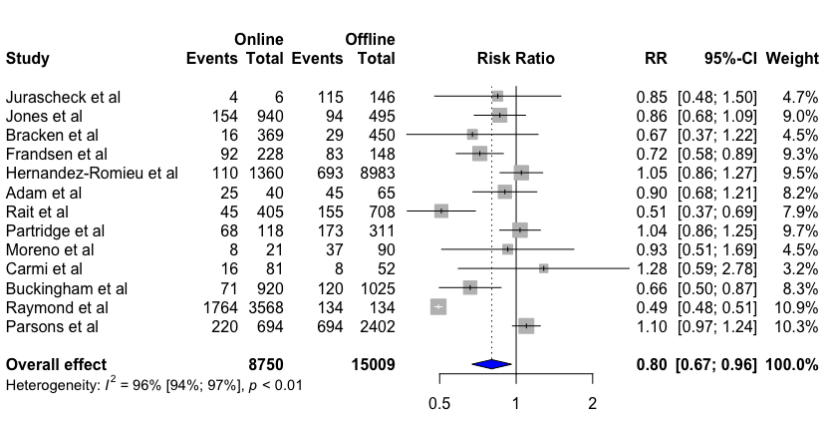
Conversion rate for online and offline recruitment. Value <1, in favor of offline recruitment; value >1, in favor of online recruitment.

For our meta-analysis on conversion rate, we included 13 of the 23 articles. We found that traditional offline recruitment strategies were superior to online recruitment strategies when comparing the percentage of participants screened who proceed to enroll into the clinical trial presented as a RR. For online recruitment, potential participants were screened through online questionnaires when clicking on online advertisements. For offline recruitment, potential participants who had shown interest in participating in the clinical trial were screened on ground or through telephone calls. Pooling the data from the 13 included studies, we found that online recruitment strategies converted significantly fewer potential participants into enrolled subjects compared with offline recruitment strategies (RR 0.8, 95% CI 0.67-0.96, *P=*.02). Hereby, offline recruitment strategies are more effective in converting participants who are screened into enrolled subjects in clinical trials. As for the recruitment rate, we found high heterogeneity between studies (*I^2^*=96%) (Figure 4).

#### Additional Analysis

##### Cost-Effectiveness of Online Recruitment

Of the 23 articles included for the meta-analyses, 13 studies reported data on the cost per enrolled participant [7,8,17,19-21,24,28,46,47,57,62,63]. The median cost per enrollee for online recruitment strategies was US $72 (range US $3.9-251.2), while the median cost per enrollee for offline recruitment strategies was US $199 (range US $19.1-839.0). However, the average cost per enrolled participant varied between the different studies, and in total, 4 out of the 13 included studies reported online recruitment to be less cost-effective compared with offline recruitment. We found a significant difference between online and offline cost-effectiveness (*P*=.048), with a V value of 17, meaning that there was a large difference between the two groups. Multimedia Appendix 3 presents cost per enrollee for all studies included in this analysis.

#### Risk of Bias for Studies Included in the Meta-Analyses

For the 14 randomized studies, none of the studies were deemed to be at risk of bias owing to allocation concealment, blinding, and missing outcome data when assessing the articles in relation to the focus of this review. Hence, no performance bias, detection bias, or attrition bias was identified. For the nine nonrandomized studies, no bias related to confounding, selection of participants, and classification of interventions was identified. However, for the studies included, the representativeness of the recruited samples was often discussed. In total, 13 of the studies included tested differences in representativeness relative to samples obtained through offline recruitment [6-8,19-21,24,28-31,36,47]. Of these, only 31% (4/13) reported that samples recruited online were similarly representative to samples recruited offline, whereas 69% (9/13) found relevant differences among the two groups. Characteristics that were most frequently reported to be imbalanced included gender (no consistent trend found), age (no consistent trend found), and education (higher education overrepresented for offline recruitment). A summary of the risk of bias can be found in Multimedia Appendix 2.

## Discussion

### Overall Findings

This study found that targeting potential participants using online remedies is an effective approach for patient recruitment for clinical research. Online recruitment strategies were superior in regard to time efficiency and cost-effectiveness compared with offline recruitment strategies. In contrast, offline recruitment strategies outperformed online recruitment strategies when examining conversion rate. To our knowledge, this is the first time a meta-analysis has been performed on the effectiveness of online patient recruitment. Our findings are consistent with findings from previous reviews on online patient recruitment [65].

### Quantitative Analysis

The recruitment rate reported in this study was only significantly better for online recruitment when days in between active recruitment were removed from the analysis (*P*=.04). This emphasizes that advertisements and campaigns on social media are efficient in a relatively short time period, after which the recruitment effectiveness starts dropping. Such findings were also reported by Juraschek et al [45], who concluded that offline recruitment was superior to online recruitment in a randomized trial recruiting cancer survivors. Therefore, online strategies should be run in intermittent campaigns to maximize full recruitment capacity, and they could also be of value when considering a short-term high-output recruitment solution. As pointed out previously, a large proportion of the studies that compared online and offline methods utilized online recruitment strategies only after realizing that offline strategies did not provide enough participants [6,7,28,36,46,62]. This could have an impact on the results for online recruitment, because the online solution here is at risk of being a short-sighted sticking-plaster solution. If online targeting had been the primary strategy of recruitment, the results might have been in the favor of online recruitment in this case [66].

Offline conversion rates were found to be significantly higher than online conversion rates (*P=*.02), as originally hypothesized. This could be due to sites having existing health records of suited patients for specific studies. For prescreening in an offline setting, it is only patients who have already shown interest in participating or referrals who already have prequalified for enrollment that are actually screened by inclusion and exclusion criteria. Further, the reach of traditional offline recruitment strategies, such as measurements of how many people read newspapers or how many people listen to campaigns on radio, cannot be quantified to the same extent as the reach for online advertisements. Hence, we do not know the actual conversion rates for specific initiatives, and therefore, the results on the conversion rate in this paper could be overestimated for offline recruitment strategies.

Our cost-effectiveness analysis showed that online recruitment is more cost-effective compared with offline recruitment. For online strategies, organic reach, such as social sharing generated online, may contribute to high cost-effectiveness, as it gives an exponential increase in message exposure, and Shere et al [6] referred to online sampling as “snow-ball recruitment,” which involves letting social media automatically expand the reach of a similar population. Even so, the cost differences arguably can be even larger, since the costs associated with offline recruitment strategies may have been underestimated. This is because for offline recruitment, the costs of personnel time were only included in very few of the cost measurements in the articles. On the other hand, setting up online advertisements and tracking and monitoring them demands personnel hours, especially in cases where the staff is inexperienced in online advertisements and campaigns and needs thorough training. As alluded earlier, to obtain the best out of online recruitment efforts, trained personnel specifically dedicated to plan proper recruitment strategies and mitigations are required. As Facebook and other online sites are in a constant state of flux, best practices for recruitment strategies here are hard to develop since no fit-for-all model works [5].

### Qualitative Analysis

This review found that the majority of published studies that used online patient recruitment were recruiting a hard-to-reach population and utilizing the potential for online recruitment tools to specifically target an underrepresented population. For this finding, it is worth noting that online remedies also have the advantage of no in-person contact, as these hard-to-reach populations often are rather stigmatized (eg, drug abusers). Further, studies included in this review targeted men and women aged 18 years or above, with no studies reporting age as a critical bottleneck for online recruitment. Overall, this study found that online recruitment was favorable compared with offline recruitment. For clinical researchers, these findings could be of great value when designing future research studies.

### Shortcomings of Online Patient Recruitment

Online methods have recruited samples with atypical demographic characteristics compared with offline methods, suggesting that the internet may reach a different population of subjects compared with samples recruited offline and hereby introduce a risk of recruitment bias. For instance, one study recruited smokers who were more likely to be nondaily smokers, exhibit high motivation to quit, and use alcohol than reported in other studies [13,67], and Bull et al [27] recruited a sample through Facebook, where the vast majority of individuals were Caucasian. On the other hand, this could introduce higher diversity in the trial, reflecting real-world demographics. Therefore, some studies argue that a combination of online and offline strategies to reach sample targets yields the most representative and unbiased samples [47]. However, during recent years, being on the internet has become the norm for almost everyone, and differences in populations may therefore be declining. Furthermore, design of advertisements is also likely an important factor, as different designs and persona character types may influence the audience signing up for a study.

### Strengths and Limitations

The vast majority of studies included in both the review and meta-analyses were RCTs, which are considered the “gold standard” for clinical trials. Further, online recruitment can be easily monitored, and data metrics from online sites are detailed and easy to obtain. However, although online recruitment metrics are widely available, many studies included in our meta-analysis lacked recruitment data for both online and offline recruitment methods. As such, the data were not complete for all of the 23 studies included in the meta-analysis. In addition, our 95% prediction intervals should be interpreted with caution because prediction intervals have been reported to be less reliable in meta-analyses with unbalanced study sizes [68]. Although speculative, more complete data could potentially have reduced any ambiguity in the results. Finally, what we have considered for our review may not be exhaustive, as there could be underlying factors that have not been investigated. We collected a limited amount of demographic data from the articles, and only touched upon the discussion related to demographics and the two recruitment methods.

### Implications for Clinical Trials

Inability to meet recruitment targets in clinical research is the biggest cause of trial delays. Our findings suggest that online strategies for patient recruitment for clinical trials can speed up recruitment and potentially reduce trial delays, as this paper substantiates that online recruitment is both more time-efficient and cost-effective compared with offline recruitment. However, this paper also suggests that to obtain the best possible results, both effort and money should be invested in online recruitment campaigns. To maximize the recruitment rate, online strategies should not be seen as an add-on to offline recruitment, but should be a primary recruitment strategy itself. Nevertheless, despite our recommendation, dealing with online recruitment methods requires engaging patients fast in the recruitment process and making sure that subjects who are transferred from the digital platform are contacted and scheduled instantly for a screening visit, as the online recruitment method may be inefficient if this is not happening [69].

### Unanswered Questions and Future Research

For future trials, researchers need to be able to adapt from an offline setting to an online setting, including online remedies in clinical trials, as hybrid and fully virtual trials are already emerging [70-72]. More research on online and offline recruitment strategies and what methods within the two recruitment strategies are most effective is therefore needed.

## Appendix

Multimedia Appendix 1 Individual characteristics of all included studies.

Multimedia Appendix 2 Individual characteristics of studies included in the meta-analyses.

Multimedia Appendix 3 Cost per enrollee for online and offline recruitment.

## Abbreviations

IR: incidence rate
IRR: incidence rate ratio
RCT: randomized controlled trial
RR: risk ratio
