# Supplementary material for: Online Patient Recruitment in Clinical Trials: Systematic Review and Meta-Analysis
Source: J Med Internet Res. 2020 Nov 4;22(11):e22179. doi: 10.2196/22179 (PMC7673977; doi:10.2196/22179)
Supplement: Multimedia Appendix 1 [file jmir_v22i11e22179_app1.pdf]

Study	Location by country	Year	Study design*	Details	Paid advertisments on social media or Google	Age	Gender	Hard-to-reach population	Conclusion	Risk of Bias*
Juraseck et al.	USA	2018	RCT	Comparing online advertising cost-effectiveness form of recruitment to traditional forms of recruitment	yes	> 18	Not specified	no	Offline recruitment superior to online	"Low risk"
Witzel et al.	UK	2019	RCT	The study uses online recruitment to recruit UK MSM and trans women who have sex with men	yes	> 16 years	Men or transgender	yes	Online recruitment was effective	"Low risk"
Morgan et al.	Australia	2013	RCT	Uses online recruitment sources for an online depression prevention intervention	yes	>18 years	Not specified	yes	Online recruitment was effective	"Low risk"
Buller et al.	USA	2012	RCT	The study compares 4 different recruitment methods, including both online and offline methods	yes	18-30 years	Not specified	no	Online and offline recruitment equally effective	"Low risk"
Schwinn et al.	USA	2017	Behavioural research trial	Uses Facebook ads and traditional paper mailings to recruit adolscent girls for a longitudinal web-based prevention trial	yes	13-14 years	Women	yes	Online recruitment was effective	"Low risk"
Cowie et al.	USA	2018	RCT	Report on the use of social media to recruit elderly people over 60 years	yes	60-78 years	Not specified	no	Online recruitment was effective	"Low risk"

Jones et al.	USA	2017	RCT	Uses Facebook to recruit young urban african american or black women for a HIV prevention trial	yes	18-29 years	Women	yes	Online recruitment was effective	"Some concerns"
Bracken et al.	Australia	2019	RCT	The study evaluates different recruitment strategies to recruit for a multi-center T2D prevention RCT	yes	50-74 years	Men	no	Offline recruitment superior to online	"Low risk"
Shere et al.	Canada	2014	RCT	Compares yearly recruitment rates in two studies, a phase 1 study using traditional recruitment methods with a phase 2study adding social media recruitment	yes	18-45 years	Women	yes	Online recruitment was effective	"Some concerns"
Frandsen et al.	Australia	2016	RCT	The study investigates how patients recruited through social media progress through a study compared to patients recruited through traditional methods	yes	>18 years	Not specified	no	Offline recruitment superior to online	"Some concerns"
Wozney et al.	Canada	2019	RCT	The study evaluates the impact and cost-effectiveness of ads for recruitment into a trial testing an Internet-based, coached intervention for parents of children with Fetal Alcohol Spectrum Disorders.	yes	18-60 years	Not specified	yes	Online recruitment was effective	"Low risk"

Graham et al.	USA	2008	Online survey	The study compares the impact of online advertising to traditional recruitment approaches for a smoking cessation intervention	no	>18 years	Not specified	yes	Online recruitment was effective	"Low risk"
Graham et al.	USA	2012	Online survey	Phase II of the study examines the effect of online ads in recruiting Latino smokers to a internet cessation intervention	no	>18 years	Not specified	yes	Online recruitment was effective	"Low risk"
Christensen et al.	Denmark	2017	Observational research trial	The study compares online and offline recruitment methods for a prospective cohort study on fertility	no	18-45 years	Women	yes	Online recruitment was effective	"Low risk"
Hernandez-Romieu et al.	USA	2014	Observational research (trial itself)	The study compares recruitment of MSM via venue-time-space sampling with Facebook recruitment	yes	>18 years	Men	yes	Online recruitment was effective	"Some concerns"
Ramo et al.	USA	2010	Online survey	The study compared three recruitment methods, including internet advertising, for young adult smokers to complete a survey about tobacco and other substance use:	yes	18-25 years	Not specified	yes	Online recruitment was effective	"Low risk"
Adam et al.	Canada	2016	RCT	The study compares efficacy of using FB paid ads recruitment of pregnant women to a RCT with traditional recruitment methods	yes	23-40 years	women	yes	Online recruitment was effective	"Some concerns"

Pedersen et al.	USA	2015	Behavioural research trial	The study utilises Facebook to recruit veterans for an online alcohol intervention study	yes	18-34 years	Not specified	yes	Online recruitment was effective	"Low risk"
Ramo et al.	USA	2014	Online survey	Uses Facebook to recruit for a smoking cessation intervention trial	yes	18-25 years	Not specified	yes	Online recruitment was effective	"Low risk"
Rait et al.	USA	2015	Observational research trial	Uses Social Media and traditional methods to recruit for a study examining the influence of metabolism on the development of nicotine addiction	yes	13-17 years	Not specified	yes	Offline recruitment superior to online	"Low risk"
Carter-Harris et al.	USA	2018	Online survey	Uses Facebook ads to recruit long-term smokers eligible for lung cancer screening for a descriptive cross-sectional survey	yes	55-77 years	Not specified	yes	Online recruitment was effective	"Low risk"
Choi et al.	Australia	2017	Behavioural research trial	The study uses Facebook ads to recruit for an online mental health study	yes	>18 years	Not specified	no	No conclusion on effectiveness	"Low risk"

Partridge et al.	Australia	2015	RCT	Compares recruitment through facebook/google ads with letters from GP and print media for a RCT on healthy lifestyle intervention for weight gain prevention in young adults	yes	18-35 years	Not specified	no	Offline recruitment superior to online	"Low risk"
Raviotta et al.	USA	2016	RCT	Compares reccruitment through Facebook ads for a RCT on immunological responses to HIV vaccine with traditional methods	yes	18-25 years	Men	yes	Online recruitment was effective	"Low risk"
Moreno et al.	USA	2017	RCT	The study compares traditional in-person recruitment methods to recruitment through social media for a physical intervention pilot study in adolescent . Inlcudes wearable tracking device (FitBit).	yes	14-18 years	Not specified	no	Online recruitment was effective	"Low risk"
Akers et al.	USA	2018	RCT	The study uses FB to recruit women for a RCT to teach support skills to female partners of male tobacco smokers	yes	25-65	women	yes	Online recruitment was effective	"Low risk"
Watson et al.	USA	2018	RCT	Uses traditional recruitment, web-based recruitment and online survey panel to recruit for a RCT smoking cessation trial	yes	>18 years	Not specified	yes	Online recruitment was effective	"Some concerns"

Carmi et al.	Israel	2014	Observational research trial	The study uses a google search based tool developed to enhance patient recruitment and compares online recruitment with print-based recruitment	no	>18 years	Not specified	no	Online recruitment was effective	"Low risk"
Buckingham et al.	USA	2017	RCT	compare the use of social media and street outreach to recruit men who have sex with men (MSM) and transgender women to a phase 2b HIV prevention vaccine trial	yes	>18 years	Not specified	yes	Online recruitment was effective	"Some concerns"
Bailey et al.	UK	2013	RCT	Adresses the feasability of amongst other online recruitment for an online RCT regarding sexually transmitted diseases	yes	16-20 years	Not specified	yes	Online recruitment was effective	"Low risk"
Munoz et al.	USA	2012	RCT (the trial itself)	illustrate that Internet interventions designed to conduct RCTs can be adapted to serve as universal health care resources	no	>18 years	Not specified	no	Online recruitment was effective	"Low risk"
Ingersoll et al.	USA	2018	Pilot RCT (the trial itself)	Uses online ads for recruitment for a pilot RCT investigating internet-delivered intervention to reduce the risk of alcohol-exposed pregnancy.	yes	18-44 years	Women	no	Online recruitment was effective	"Low risk"

Buller et al.	Canada	2015	RCT (the trial itself)	Patients were recruited through online promotions nationwide for a RCT evaluating the effect of a smartphone sun-safe application	yes	>18 years	Not specified	no	no conclusion on effectiveness	"Low risk"
Schaub et al.	Switzerland	2012	RCT (the trial itself)	Uses both online and offline media to recruit for a web-based RCT investigating an intervention to reduce cocain consumption in problematic cocain users	yes	>18 years	Not specified	yes	no conclusion on effectiveness	"Low risk"
Heckmann et al.	USA	2017	RCT (the trial itself)	Uses online recruitment for an online intervention on skin cancer risk reduction in young people	no	18-25 years	Not specified	no	no conclusion on effectiveness	"Low risk"
Van Lettow et al.	Netherland	2015	RCT (the trial itself)	Uses both online and traditional methods to recruit for an online RCT on an intervention on alcohol consumption	no	>18 years	Not specified	yes	no conclusion on effectiveness	"Low risk"
Cobb et al.	USA	2016	RCT (the trial itself)	Smoking cessation trial conducted entirely through Facebook	yes	>18 years	Not specified	yes	Online recruitment was effective	"Low risk"
Irvine et al.	USA	2013	RCT (the trial itself)	Uses online recruitment for an internet intervention to promoto physical activity by sedentary older adults	yes	>55 years	Not specified	yes	no conclusion on effectiveness	"Low risk"

Kuhn et al.	USA	2017	RCT (the trial itself)	Uses online ads to recruit for a RCT of a smartphone app for PTSD symptoms	yes	>18 years	Not specified	yes	no conclusion on effectiveness	"Low risk"
Jonas B et al.	Germany	2016	RCT (the trial itself)	Uses online and in-house recruitment for an internet-based intervention for burnout in the German population	yes	>18 years	Not specified	no	no conclusion on effectiveness	"Low risk"
Gordon et al.	USA	2006	RCT	Report evaulating recruitment methods (online and traditional) for a RCT on a web-based program for smoking cessation	yes	>18 years	Not specified	yes	Online recruitment was effective	"Some concerns"
Bull et al.	USA	2004	Online survey	Evaluates recruitment rates in an internet-based RCT to increase STD prevention in MSM	no	>18 years	Men	yes	Online recruitment was effective	"Low risk"
Bull et al.	USA	2008	RCT	Presents recruitment findings for an online RCT on HIV prevention	no	15-25 years	Men	yes	Online recruitment was effective	"Low risk"
Das et al.	Australia	2017	Observational research trial	Using Facebook to Recruit Young Australian Men Into a Cross-Sectional Human Papillomavirus Study	yes	18-35 years	Men	no	Online recruitment was effective	"Low risk"

Anguera et al.	USA	2015	RCT (the trial itself)	Uses traditional, social media, and search-engine recruitment for a fully mobile RCT on depression	yes	>18 years	Not specified	yes	Online recruitment was effective	"Low risk"
Bricker et al.	USA	2013	Pilot RCT (the trial itself)	Uses both online and traditional methods to recruit for an online RCT on a smoking cessation intervention	yes	>18 years	Not specified	yes	no conclusion on effectiveness	"Low risk"
Brodar et al.	USA	2016	RCT	Uses both online and traditional methods to identify diverse smokers	yes	>18 years	Not specified	yes	Online recruitment was effective	"Some concerns"
Raymond et al.	USA	2009	RCT	Compares remote and traditional recruitment for sampling MSM in San Francisco	yes	>18 years	Men	yes	Online recruitment was effective	"Some concerns"
Parsons et al.	USA	2012	Behavioural research trial	Compares remote and traditional recruitment for sampling MSM for a behavioural trial	yes	>18 years	Men	yes	Online recruitment was effective	"Some concerns"
Heffner et al.	USA	2013	RCT	Compare recruitment methods for web-based intervention on tobacco use	yes	>18 years	Not specified	yes	Online recruitment was effective	"Some concerns"
Cobb et al.	USA	2014	RCT	Recruitment through Facebook ads for an internet-based RCT on smoking cessation conducted through an app	yes	>18 years	not specified	yes	Online recruitment was effective	"Low risk"

Maloni et al.	USA	2013	Observational research (trial itself)	Exploratory study on feasibility of social media recruiting of women with postpartum depression after pregnancy complication.	no	>18 years	women	yes	Online recruitment was effective	"Low risk"
Bowen et al.	USA	2004	RCT	Compares traditional and remote recruitment methods for sampling rural MSM for a HIV risk trial	no	>18 years	Men	yes	Online recruitment was effective	"Low risk"
MacDonnel et al	USA	2019	Behavioural research trial	Uses ads on social media including FB to recruit a hidden population of women for an alcohol-exposed pregnancy risk reduction study	yes	>18 years	women	yes	Online recruitment was effective	"Low risk"
Prescott et al	USA	2016	Prevention study	Reaches young, gay, bisexual and queer men online thorugh social media advertising including Facebook campaigns	yes	14-18 years	Men	yes	Online recruitment was effective	"Low risk"
Yuan et al	USA	2014	Cross-sectional survey	Uses social media advertisements to recruit HIV-positive participants for a cross-sectional survey	yes	>18 years	Men	yes	Online recruitment was effective	"Low risk"
Clarke et al	Australia	2019	RCT	Uses Google and Facebook to recruit for a web-based therapy to improve social functioning in adults with type 2 Diabetes mellitus.	yes	>18 years	not specified	no	no conclusion on effectiveness	"Low risk"

Byaruhanga et al	Australia	2019	Observational research study	Compares cost per participants for recruiting for a smoking cessation trial thorugh online and offline recruitment methods	yes	>18 years	not specified	yes	Not clarified	"Some concerns"
Guthrie et al	USA	2019	Pilot study itself	Uses Facebook to recruit menopausal women with bothersome vaginal symptoms	yes	50-70 years	women	yes	Online recruitment was effective	"Low risk"
Williams et al	USA	2012	Behavioural research trial	Uses both online and offline recruitment strategies african americans for an OCD study	yes	>18 years	not specified	yes	no conclusion on effectiveness	"Low risk"
Pratap et al	USA	2018	RCT	Uses internet-based advertisements to recruit for a fully remote RCT on a depression intervention	yes	>18 years	not specified	yes	no conclusion on effectiveness	"Low risk"
