# Supplementary material for: Online Patient Recruitment in Clinical Trials: Systematic Review and Meta-Analysis
Source: J Med Internet Res. 2020 Nov 4;22(11):e22179. doi: 10.2196/22179 (PMC7673977; doi:10.2196/22179)
Supplement: Multimedia Appendix 2 [file jmir_v22i11e22179_app2.pdf]

Study	Location by country	Year	Study design*	Enrollment rate months (online) No. patients enrolled per month	Enrollment rate months (offline) No. patients enrolled per month	Enrollment rate active campaign days (online) No. patients enrolled per day	Enrollment rate active days (offline) No. patients enrolled per day	Conversion rate (online)	Conversion rate (offline)
Juraseck et al.	USA	2018	RCT	0,7	6,4	NA	NA	67%	78%
Jones et al.	USA	2017	RCT	22	13,4	0,8	0,5	16%	19%
Bracken et al.	Australia	2019	RCT	0,5	0,6	0,1	0,02	2%	6%
Frandsen et al.	Australia	2016	RCT	6,6	5,9	NA	NA	40%	60%
Hernandez-Romieu et al.	USA	2014	Observational research (trial itself)	4,8	23,1	NA	NA	8%	8%
Adam et al.	Canada	2016	RCT	25	6,4	0,9	0,2	63%	69%
Rait et al.	USA	2015	Observational research trial	1,5	5,2	NA	NA	11%	22%
Partridge et al.	Australia	2015	RCT	5,6	8,7	1,5	0,3	58%	56%

Moreno et al.	USA	2017	RCT	1,6	7,5	NA	NA	38%	41%
Watson et al.	USA	2018	RCT	148,5	37,3	NA	NA	NA	NA
Carmi et al.	Israel	2014	Observational research trial	1,6	0,8	NA	NA	20%	15%
Buckingham et al.	USA	2017	RCT	2,2	2,6	NA	NA	8%	12%
Gordon et al.	USA	2006	RCT	58,7	109,5	NA	NA	NA	NA
Bull et al.	USA	2004	Online survey	56,8	36,7	4	1,9	NA	NA
Anguera et al.	USA	2015	RCT (the trial itself)	64,4	520,2	NA	NA	NA	NA
Brodar et al.	USA	2016	RCT	79,3	92	NA	NA	NA	NA
Raymond et al.	USA	2009	RCT	147	11,2	31,5	0,4	49%	100%

Parsons et al.	USA	2012	Behavioural research trial	169,3	115,7	6,3	4,3	32%	30%
Heffner et al.	USA	2013	RCT	47,7	26,7	NA	NA	NA	NA
Christensen et al	Denmark	2017	Observational research trial	30,5	10,3	NA	NA	NA	NA
Shere et al	Canada	2014	RCT	6,4	0,8	NA	NA	NA	NA
Byaruhanga et al	Australia	2019	Observational research trial	34,2	1	NA	NA	NA	NA
William et al	USA	2012	Behavioural research trial	4,1	6,5	NA	NA	NA	NA
