# Supplementary material for: Online Patient Recruitment in Clinical Trials: Systematic Review and Meta-Analysis
Source: J Med Internet Res. 2020 Nov 4;22(11):e22179. doi: 10.2196/22179 (PMC7673977; doi:10.2196/22179)
Supplement: Multimedia Appendix 3 [file jmir_v22i11e22179_app3.pdf]

Study	Cost per enrollee (online)	Cost per enrollee (offline)
Jones et al.	66.46 USD	149.62 USD
Bracken et al.	251.2 USD	794.7 USD
Frandsen et al.	36.7 USD	34.1 USD
Hernandez-Romieu et al.	68.6 USD	91.2 USD
Rait et al.	149.64 USD	43.78 USD
Partridge et al.	23.9 USD	84 USD
Moreno et al.	41 USD	19.1 USD
Watson et al.	31.89 USD	20.3 USD
Gordon et al.	3.9 USD	134.8 USD
Brodar et al.	8.3 USD	23.2 USD
Heffner et al.	32.2 USD	105.3 USD
Byaruhanga et al	41.26 USD	839 USD

Williams et al	179.29 USD	253 USD
**Total Average costs**	**72 USD**	**199 USD**
